# The Fatty Acid Profile and Oxidative Stability of Meat from Turkeys Fed Diets Enriched with n-3 Polyunsaturated Fatty Acids and Dried Fruit Pomaces as a Source of Polyphenols

**DOI:** 10.1371/journal.pone.0170074

**Published:** 2017-01-11

**Authors:** Jerzy Juskiewicz, Jan Jankowski, Henryk Zielinski, Zenon Zdunczyk, Dariusz Mikulski, Zofia Antoszkiewicz, Monika Kosmala, Przemyslaw Zdunczyk

**Affiliations:** 1 Institute of Animal Reproduction and Food Research, Polish Academy of Sciences, Olsztyn, Poland; 2 Department of Poultry Science, University of Warmia and Mazury, Olsztyn, Poland; 3 Department of Animal Nutrition and Food Management, University of Warmia and Mazury, Olsztyn, Poland; 4 Institute of Food Technology and Analysis, Lodz University of Technology, Lodz, Poland; INIA, SPAIN

## Abstract

The aim of this study was to determine the efficacy of different dietary fruit pomaces in reducing lipid oxidation in the meat of turkeys fed diets with a high content of n-3 polyunsaturated fatty acids (PUFAs). Over a period of 4 weeks before slaughter, turkeys were fed diets with the addition of 5% dried apple, blackcurrant, strawberry and seedless strawberry pomaces (groups AP, BP, SP and SSP, respectively) and 2.5% linseed oil. Pomaces differed in the content (from 5.5 in AP to 43.1 mg/g in SSP) and composition of polyphenols Proanthocyanidins were the main polyphenolic fraction in all pomaces, AP contained flavone glycosides and dihydrochalcones, BP contained anthocyanins, and SP and SSP—ellagitannins. The n-6/n-3 PUFA ratio in all diets was comparable and lower than 2:1. In comparison with groups C and AP, the percentage of n-3 PUFAs in the total fatty acid pool of white meat from the breast muscles of turkeys in groups BP, SP and SSP was significantly higher, proportionally to the higher content of α-linolenic acid in berry pomaces. The fatty acid profile of dark meat from thigh muscles, including the n-6/n-3 PUFA ratio, was similar and lower than 3:1 in all groups. Vitamin A levels in raw breast muscles were higher in group AP than in groups C and BP (*P*<0.05). The addition of fruit pomaces to turkey diets lowered vitamin E concentrations (*P* = 0.001) in raw breast muscles relative to group C. Diets supplemented with fruit pomaces significantly lowered the concentration of thiobarbituric acid reactive substances (TBARS) in raw, frozen and cooked meat. Our results indicate that the dietary application of dried fruit pomaces increases the oxidative stability of meat from turkeys fed linseed oil, and strawberry pomace exerted the most desirable effects due to its highest polyphenol content and antioxidant potential.

## Introduction

High consumption of meat and meat products in developed countries is one of the factors responsible for a high and undesirable ratio of n-6 to n-3 polyunsaturated fatty acids (PUFAs) in excess of 10:1 [1]. This applies also to poultry products, which account for more than 40% of consumed meat [2]. For this reason, an increase in the content of n-3 PUFAs in meat, which leads to an improvement in the n-6/n-3 PUFA ratio in the human diet, is an important consideration in the process of adding value to poultry products for the health conscious consumer [3]. This goal can be achieved by supplementing poultry diets with oils rich in n-3 PUFAs, such as linseed oil [4].

The inhibition of oxidative processes in the dietary lipid fraction and in muscle lipids poses a key problem in the supplementation of poultry diets with vegetable oils [5]. To address this issue, poultry diets are commonly enriched with vitamin E and selenium [6], and the search for new, natural antioxidants, such as polyphenol extracts, continues [7, 8]. Selected polyphenols exhibit powerful antioxidant effects, and they are added to poultry diets to enhance the beneficial influence of vitamin E and offer greater antioxidant protection for birds and poultry products [9, 10, 11].

The supplementation of poultry diets with flavonoid extracts increased vitamin E concentrations and decreased the content of malondialdehyde (MDA) in the blood serum of quails and broiler chickens [9, 10]. The addition of grapeseed extract produced antioxidant effects in broiler diets and excreta [12]. In another experiment, grapeseed, rosemary and green tea extracts were not highly effective in inhibiting lipid oxidation in the blood of broilers [13], which could suggest that the physiological effects of polyphenols are influenced by their source, method of acquisition and concentrations. The extraction of pure polyphenols requires complex methods involving alcohol or acetone, which increases the costs of the extraction process.

Pomaces from the production of grape juice are a rich source of polyphenols which increased antioxidant activity in poultry diets and excreta, inhibited the oxidation of lipids in body tissues [14] and demonstrated similar antioxidant effects to vitamin E [15]. In poultry nutrition, similar effects are delivered by other by-products of fruit processing, including apples whose global annual production reaches 75 million tons [16]. Apple pomace had a positive influence on blood parameters in pigs [17], but there are no published studies documenting the effect of apple pomace on poultry health. The antioxidant effects of strawberry pomace were also observed in a study of rats [18].

In Poland, annual strawberry production is estimated at 170,000 tons [19], which implies that strawberry pomace can be widely used in animal nutrition. Strawberry and blackcurrant pomaces are rich in polyphenols, and they increase the antioxidant capacity of poultry diets, which could be particularly important in diets with a higher content of vegetable oils.

The objective of this study was to determine whether the inclusion of 5% dried fruit pomaces (apple, blackcurrant and strawberry pomaces with various degree of processing) as a source of polyphenols in turkey diets can increase the antioxidant capacity of diets and limit oxidative changes in meat with a lowered n-6/n-3 PUFA ratio.

## Materials and Methods

### Experimental design and diet composition

The experiment was carried out at the Research Laboratory of the Department of Poultry Science, University of Warmia and Mazury in Olsztyn (Poland). All experimental procedures were approved by the Local Animal Care and Use Committee (Olsztyn, Poland), and the study was carried out in accordance with EU Directive 2010/63/EU for animal experiments. The study was performed on 525 Big 7 female turkeys aged 1 day to 15 weeks, divided into 5 groups of 105 birds each. Each group was kept in 7 pens of 15 birds per pen (7 replicates per group).

The experimental diets differed in their content of dried apple pomace (AP), dried blackcurrant pomace (BP), dried strawberry pomace (SP) and dried seedless strawberry pomace (SSP) as an additional source of dietary polyphenols. The diets fed to control group (C) turkeys contained cellulose to level out their crude fiber content. The chemical composition of diets and different pomace drying methods (AP, BP and SP were dried by convection in the SB-1.5 drum dryer, whereas SSP was vacuum dried at a temperature of 70°C) are presented elsewhere [20].

The composition of diets fed to turkeys aged 11–15 weeks is presented in Table 1. The diets fed to birds aged 1–10 weeks also contained 5% dried fruit pomaces and only soybean oil (without linseed oil) to level out their energy value. In the last feeding phase, diets were supplemented with linseed oil to increase the content of n-3 PUFAs that are more susceptible to oxidation. All diets contained 0.5% of the commercial Extramix premix which provided equal amounts of vitamins, including vitamin A (all-trans-retinol acetate) - 13 000 IU, vitamin E (all-rac-α-tocopheryl acetate) - 40 mg and organic Se– 0.3 mg/kg. The level of vitamin E supplementation was substantially higher than that recommended by the NRC [21] for turkeys fed standard commercial diets, but lower than that applied in our earlier experiment [22] where vitamin E added at 54 mg/kg of diet had no protective effect on the oxidative stability of meat in turkeys fed diets containing linseed oil.

**Table 1:** Composition and nutritional value of diets fed to turkeys at 11–15 weeks of age.

	Experimental diet^1^				
	C	AP	BP	SP	SSP
Component (%)					
Wheat	58.41	55.21	57.48	57.23	57.23
Soybean meal	30.83	31.03	29.40	29.34	29.34
Vitacel^®^ cellulose^1^	2.33	-	-	-	-
Fruit pomace^1^	-	5.00	5.00	5.00	5.00
Soybean oil	2.84	3.17	2.52	2.84	2.84
Linseed oil	2.50	2.50	2.50	2.50	2.50
Sodium bicarbonate	0.10	0.10	0.10	0.10	0.10
Fodder salt	0.14	0.14	0.14	0.14	0.14
Limestone	1.23	1.23	1.23	1.23	1.23
Monocalcium phosphate	0.55	0.575	0.57	0.55	0.55
Ronozyme P and WX	0.03	0.03	0.03	0.03	0.03
DL-methionine 99%	0.18	0.18	0.18	0.18	0.18
L-lysine 99%	0.29	0.28	0.28	0.29	0.29
L-threonine	0.07	0.07	0.07	0.07	0.07
Premix^2^	0.50	0.50	0.50	0.50	0.50
Nutritional value (%)					
Total protein	21.5	21.5	21.5	21.5	21.5
Crude fat	6.82	7.21	7.16	7.05	7.05
Crude fiber	3.96	3.96	3.94	4.08	4.06
ME, MJ/kg	12.6	12.6	12.6	12.6	12.6
Lysine	1.25	1.25	1.25	1.25	1.25
Methionine	0.48	0.48	0.48	0.48	0.48
Met + Cys	0.86	0.86	0.86	0.85	0.85
Threonine	0.86	0.80	0.80	0.80	0.80
Ca	0.75	0.75	0.75	0.75	0.75
P	0.30	0.30	0.30	0.30	0.30
Na	0.10	0.10	0.10	0.10	0.10

^1^Dietary treatments with addition of: C–cellulose, AP–apple pomace, BP—blackcurrant pomace, SP—strawberry pomace, SSP–seedless strawberry pomace.

^2^0.5% of the Premix provided per kg of diet: all trans-retinol acetate—13000 IU, cholecalciferol—3000 IU, all-rac-α-tocopheryl acetate—40 mg, vitamin K3−2 mg, vitamin B1−2 mg, vitamin B2−8 mg, vitamin B6−3.5 mg, niacin—65 mg, pantothenic acid—18 mg, folic acid—1.5 mg, biotin—0.2 mg, choline chloride—400 mg, Mn—100 mg, Zn—80 mg, Fe—50 mg, Cu—8 mg, I—0.8 mg, Se—0.3 mg.

### Analysis of the content and composition of polyphenols in fruit pomaces and diets

The total phenolic content of turkey diets was determined using the Folin-Ciocalteu reagent, as described by Singleton et al. (1999) [23], and was expressed in mg of gallic acid equivalents (GAE) per gram of diet. The composition of polyphenols in fruit pomaces was determined by HPLC after extraction with acetone in triplicate. A ground analytical sample of 0.5 g was placed in a test tube, and 4 ml of 70% acetone solution was added. The mixture was sonified at 22^°^C for 15 minutes, next the solution was centrifuged (4800 × *g*) and transferred to a flask. The above procedure was performed in two replications, and the extracts were combined. Acetone was distilled in a rotary vacuum evaporator, and dry residues were dissolved in 2 ml of 70% glycerol. The extract was analyzed in a HPLC system with a DAD detector (Dionex, Sunnyvale, CA, USA), on a Gemini 5u C18 110A-2509 column, 4.60 mm (Phenomenex, Torrance, CA, USA). Phase A involved 0.05% phosphoric acid solution, and phase B– 0.05% solution of phosphoric acid in acetonitrile. The applied gradient had a flow rate of 1.25 ml/min: 5 min of stabilization with 4% B, followed by 5 to 12.5 min with 4–15% B, 12.50 to 42.40 min with 15–40% B, 42.40 to 51.80 min with 40–50% B, 51.80 to 53.40 min with 50% B, and 53.40 to 55 min with 4% B. Column temperature was 25°C. The analyzed compounds were identified with commercial standards supplied by Extrasynthese, Genay (France) and Sigma-Aldrich (St. Louis, USA). The proanthocyanidin content of fruit pomaces was determined in a separate procedure described by Kennedy and Jones [24]. The polyphenol compositional analysis was described in detail in another study [25].

### Analysis of the antioxidant potential of fruit pomaces and experimental diets

The antioxidant activity of fruit pomaces was measured directly in the 2,2-diphenyl-1-picrylhydrazyl (DPPH^•^) assay according to the method of Hatano [26], and the antioxidant activity of experimental diets was determined in accordance with the method described by Zielińska *et al*. [27]. The antioxidant activity of diets was also measured in the ABTS^•+^ assay in line with the method proposed by Re *et al*. [28] and the photochemiluminescence assay against superoxide anion radical (^•^ O_2_^-^) according to the method of Popov and Lewin [29]. Measurements were carried out in a temperature-controlled UV-160 1PC spectrophotometer with a CPS-Controller (Shimadzu, Tokyo, Japan), and the results were expressed in μmol Trolox/g of sample. The photochemiluminescence assay was used to determine total antioxidant capacity as the sum of the antioxidant potentials of hydrophilic (ACW) and lipophilic (ACL) fractions. The ACL fraction was extracted from samples with 80% methanol, followed by a mixture of methanol and hexane (4:1, v/v). Measurements were performed in a Photochem^®^ apparatus with ACW and ACL analytical kits supplied by Analytik Jena (Leipzig, Germany).

### Collection, storage and preparation of meat samples for analysis

At 15 weeks of age, seven birds representing the average body weight of each group were selected for blood collection (blood was collected from the wing vein into sterile tubes containing EDTA), and slaughtered in a processing plant 8 h after feed withdrawal (the Faculty of Animal Bioengineering’s slaughterhouse, the University of Warmia and Mazury in Olsztyn). The equipment of the slaughterhouse and all applied procedures gained approval of the Local Animal Care and Use Committee (Olsztyn, Poland; permission number 78/2012/N). The birds were electrically stunned (400 mA, 350 Hz), hung on a shackle line and exsanguinated by a unilateral neck cut severing the right carotid artery and jugular vein. Turkeys were scalded at 61°C for 60 s, defeathered in a rotary drum picker for 25 s, and manually eviscerated. As described previously [30], the following indicators of blood plasma antioxidant status were determined: ascorbic acid concentration, the activities of catalase and superoxide dismutase (SOD), and the ferric reducing ability of plasma (FRAP).

Carcasses were cooled at a temperature of 12°C for 30 minutes and stored at a temperature of 4°C. After 24 h, the left breast muscle and the left thigh were sampled for analyses. A portion of the samples was vacuum packaged, frozen at -20°C and stored for 3 months until analyses of thiobarbituric acid reactive substances (TBARS). The content of TBARS, retinol and α-tocopherol was determined in raw meat, frozen meat and cooked fresh meat and cooked frozen meat. Meat samples were cooked in a steam and convection oven (BECK FCV 4 EDS GmbH Jagsthausen, Germany) for 30 minutes until the temperature inside muscles reached 75°C.

### Analysis of the fatty acid profile, antioxidant status, and color parameters of meat

The fatty acid profile of diets and meat was determined in samples extracted with a mixture of chloroform and methanol (2:1, v:v), esterified by Peisker’s method (1964) [31], and subjected to gas chromatography in a 6890 N gas chromatograph (Agilent Technologies Inc., Palo Alto, CA) equipped with a flame ionization detector (FID). Column (capillary, 0.32 mm x 30), injector and detector temperatures were set at 180, 225 and 250°C, respectively. Helium was applied as a carrier gas at a flow rate of 0.7 cm^3^/min. Fatty acids were identified based on their retention times and were expressed as the percentage of total identified fatty acids. All analyses were carried out in duplicate.

Tocopherol and retinol concentrations were measured by HPLC (Shimadzu, Japan), according to the method described by Rettenmaier and Schüep (1992) [32], with the use of rac-α-tocopherol (Sigma, Switzerland) as the reference standard. Changes in the oxidative status of lipids were determined based on TBARS levels, measured by the method proposed by Draper and Hadley (1990) [33]. Absorbance was measured with the SPECORD 40 spectrophotometer (Analityk Jena AG, Germany), and TBARS levels were expressed in nmol of malondialdehyde (MDA) per gram of meat.

The pH of meat (breast muscle) was measured 24 h after carcass chilling (Testo 206–pH2 pH-meter, Testo AG, Lenzkirch, Germany). Hunter L* (lightness), a* (redness), and b* (yellowness) values were determined in breast muscle samples using the MiniScan XE Plus color difference meter (Hunter Associates Laboratory Inc., Reston, VA, USA). The average of three readings taken from the cross-section of the muscle free from color defects, bruising and hemorrhages was recorded.

### Statistical analysis

The results were analyzed statistically using the GLM procedure in Statistica 8.0PL software. Differences were regarded as significant at p ≤ 0.05. All data were expressed as mean values with standard error of the mean (SEM).

## Results

The inclusion of fruit pomaces did not affect the nutritional value of turkey diets, including metabolizable energy content and the content of crude protein and major amino acids which were leveled out by minor modifications in the proportions of the main components (soybean meal and wheat) and the addition of soybean oil (Table 1). The crude fiber content of all diets approximated 4%.

The analyzed fruit pomaces differed in their content and composition of polyphenols (Table 2). In all pomaces, proanthocyanidins were the main polyphenolic fraction. The lowest concentration of polyphenols was noted in apple pomace (AP, 5.5 mg/g in HPLC analysis) which contained proanthocyanidins (4.5 mg/g) and similar amounts of flavone glycosides (myricetins, quercetins and kaempferol; 0.5 mg/g) and dihydrochalcones (phloridzin and phloretin; 0.5 mg/g). The polyphenol content of BP was more than 5-fold higher (31.0 mg/g) in comparison with AP, and in addition to proanthocyanidins (26.0 mg/g), BP also contained anthocyanins (4.9 mg/g) and trace amounts of flavone glycosides. The polyphenolic fraction of strawberry pomace was composed of proanthocyanidins and ellagitannin, and the content of ellagitannin and total polyphenols in SSP was more than 4 times higher than in SP (43.1 and 10.3 mg/g, respectively). Considerable differences in the polyphenolic fraction of fruit pomaces were reflected in the analyzed values of the antioxidant activity of pomaces, ranging from the lowest value in AP (32 μmol TE/g) to the highest value in SSP (256 μmol TE/g).

**Table 2:** Composition of the polyphenolic fraction in fruit pomaces (mg/g) and the antioxidant activity of pomaces (μmol TE/g; using the 2,2-diphenylpicrylhydrazyl (DPPH) assay).

	Apple (AP)	Blackcurrant (BP)	Strawberry ^1^ (SP)	Seedless strawberry ^2^ (SSP)
Flavone glycosides^3^	0.51	0.17	0.99	1.60
Anthocyanins	-	4.87	-	0.90
Phenolic acids^4^	-	-	-	0.94
Dihydrochalcones^5^	0.49	-	-	0.05
Ellagitannins	-	-	4.24	15.4
Proanthocyanidins	4.50	26.0	5.10	24.2
Total polyphenols	5.50	31.04	10.33	43.09
Antioxidant activity	32.0	102.8	84.7	256.4

^1^Unsorted native pomaces dried in a drum dryer.

^2^Seedless pomace fraction, vacuum dried.

^3^Myricetin, quercetin and kaempferol glycosides.

^4^ Sum of *p*-coumaric and *p*-benzoic acids.

^5^ Sum of phloridzin and phloretin.

The experimental diets differed in their content of phenolic compounds, which was proportional to polyphenol concentrations in fruit pomaces (Table 3). In comparison with the control diet, the lowest increase in polyphenol levels (by 0.18 mg/g) was noted in AP diets. Polyphenol concentrations increased by 0.21 mg/g in BP and SP diets, and by 0.31 mg/g in the SSP diet, which affected the antioxidant activity of experimental diets. In comparison with the control diet, the lowest DPPH and ABTS radical scavenging effects were noted in the AP diet, and the highest–in the SSP diet. The results of chemiluminescence assays revealed that the ACW fraction was the major contributor to the antioxidant capacity of diets, whereas the ACL fraction played a less important role. In comparison with the control diet, the lowest increase in the antioxidant capacity of the ACW fraction was observed in group AP (from 0.71 to 1.24 μmol TE/g), whereas the highest increase was noted in group SSP (to 1.73 μmol TE/g). The total antioxidant capacity of SP and SSP diets was at least two-fold higher relative to the control diet.

**Table 3:** The polyphenol content and antioxidant capacity of diets containing cellulose or various fruit pomaces.

	Experimental group^1^				
	C	AP	BP	SP	SSP
Polyphenol content FC^2^ of diets, mg/g	1.27	1.45	1.48	1.48	1.58
Antioxidant activity of diets					
DPPH, μmol TE/g	1.33	1.65	1.91	1.93	2.16
ABTS, μmol TE/g	7.71	7.89	8.18	8.23	9.55
Antioxidant capacity of diets					
lipophilic extract, μmol TE/g	0.29	0.36	0.48	0.58	0.48
hydrophilic extract, μmol TE/g	0.71	1.24	1.31	1.42	1.73
total capacity, μmol TE/g	1.00	1.60	1.79	2.00	2.21

^1^Dietary treatments with the addition of: C–cellulose, AP–apple pomace, BP—blackcurrant pomace, SP—strawberry pomace, SSP–seedless strawberry pomace.

^2^Polyphenol content was measured with the use of the Folin-Ciocalteu reagent.

The compared fruit pomaces differed in their crude fat content and fatty acid profile (Table 4). In comparison with AP, crude fat content and the percentage of α-linolenic acid in total fatty acids were higher in BP, SP and SSP. The n-6/n-3 PUFA ratio was highly differentiated, ranging from nearly 12:1 in AP to 3:1 in BP and below 2:1 in SP and SSP. In comparison with the control diet, AP and BP diets were characterized by similar concentrations of linoleic acid (38.3–39.6%) and α-linolenic acid (19.6–20.5%) as well as a similar n-6/n-3 PUFA ratio at 1.9:1 (Table 5). SP and SSP diets were somewhat more abundant in α-linolenic acid (21.5–22.8%) and had a somewhat lower n-6/n-3 PUFA ratio (1.8:1).

**Table 4:** Crude fat content and fatty acid profile of various fruit pomaces (% of total fatty acids).

	Fruit pomace^1^			
	AP	BP	SP	SSP
Crude fat	2.63	13.8	10.4	9.64
Fatty acids				
C12:0	0.19	0.04	0.07	0.03
C12:1	0.40	0.07	0.03	0.06
C14:0	0.42	0.12	0.18	0.19
C15:0	0.09	0.05	0.05	0.06
C16:0	10.2	8.92	7.11	6.60
C16:1	0.13	0.13	0.22	0.23
C17:0	0.23	0.11	0.13	0.03
C17:1	0.08	0.06	0.06	0.02
C18:0	2.68	2.03	1.81	1.60
C18:1 Cis9	21.3	15.6	14.2	16.7
C18:2 n-6	47.8	53.6	47.5	44.0
C18:3 n-3	4.91	17.9	27.3	29.0
C20:0	1.43	0.46	0.96	1.34
C20:2 n-6	9.49	0.37	0.09	0.04
C20:4 n-6	0.71	0.44	0.34	0.03
n-6/n-3 PUFA ratio	12:1	3.0:1	1.7:1	1.5:1

^1^AP–apple pomace, BP—blackcurrant pomace, SP—strawberry pomace, SSP–seedless strawberry pomace.

**Table 5:** Fatty acid profile of experimental diets, % of total fatty acids.

FA	Diet^1^				
	C	AP	BP	SP	SSP
C12:0	0.03	0.00	0.00	0.00	0.00
C14:0	0.09	0.10	0.09	0.08	0.09
C15:0	0.05	0.05	0.05	0.03	0.05
C16:0	12.6	13.6	13.2	10.7	12.6
C16:1	0.14	0.14	0.13	0.11	0.14
C17:0	0.15	0.20	0.22	0.10	0.17
C17:1	0.06	0.07	0.07	0.06	0.06
C18:0	4.05	4.24	4.21	3.68	4.04
C18:1 Cis9	21.6	22.6	22.4	19.9	213
C18:2 n-6	39.6	38.3	38.3	41.6	39.0
C18:3 n-3	20.5	19.7	19.9	22.8	21.5
C20:0	0.30	0.33	0.43	0.28	0.34
C20:1	0.25	0.26	0.26	0.23	0.27
C20:2 n-6	0.09	0.04	0.19	0.04	0.05
C20:4 n-6	0.06	0.03	0.09	0.08	0.05
C22:0	0.33	0.36	0.36	0.32	0.35
C22:6 n-3 DHA	0.15	0.06	0.10	0.09	0.04
SFA	17.6	18.9	18.6	15.2	17.7
UFA	82.4	81.1	81.4	84.8	82.3
MUFA	22.0	23.1	22.9	20.3	21.7
PUFA	60.4	58.1	58.6	64.6	60.6
n-3 PUFA	20.7	19.7	20.0	22.9	21.5
n-6 PUFA	39.8	38.4	38.6	41.7	39.1
n-6/n-3 PUFA ratio	1.9:1	1.9:1	1.9:1	1.8:1	1.8:1

^1^Dietary treatments with the addition of: C–cellulose, AP–apple pomace, BP—blackcurrant pomace, SP—strawberry pomace, SSP–seedless strawberry pomace.

No significant differences in the final body weights of birds or the yields of breast and thigh muscles in the carcass were observed between experimental groups (Table 6). Breast muscle yield approximated 24%, and thigh muscle yield exceeded 10%. No significant differences were noted in the pH_24h_ or color parameters of fresh breast muscle.

**Table 6:** Final body weights of turkeys, the yields of breast and thigh muscles in the carcass, and breast meat color parameters.

	Experimental group^1^					SEM	*P*
	C	AP	BP	SP	SSP		
Final body weight, kg	10.4	10.6	10.5	10.4	10.6	0.045	0.779
Thigh muscles yield, %	10.3	10.0	10.8	10.4	10.6	0.740	0.287
Breast meat:							
Muscle yield, %	23.6	23.6	24.2	24.1	24.2	1.591	0.880
pH_24h_	5.67	5.70	5.88	5.72	5.69	0.213	0.313
L^*^	51.6	52.6	52.8	52.4	52.7	2.141	0.838
a^*^	5.21	5.72	5.14	5.44	4.84	0.984	0.461
b^*^	10.5	11.0	11.2	10.8	10.6	0.960	0.574

^1^Dietary treatments with the addition of: C–cellulose, AP–apple pomace, BP—blackcurrant pomace, SP—strawberry pomace, SSP–seedless strawberry pomace. Values are means of 7 observations per treatment. L*—lightness, a*—redness, b*—yellowness.

SEM–standard error of the mean.

Breast meat differed in the percentage of α-linolenic acid in total fatty acids, and significant differences were noted between groups C and AP vs. groups BP, SP and SSP (P = 0.05) (Table 7). No significant differences were observed in the concentrations of total saturated fatty acids, monounsaturated fatty acids and total n-6 PUFAs. In breast muscles, the highest n-6/n-3 PUFA ratio was determined in group AP (P<0.05 vs. the remaining groups), and a significant difference was found between group C and group SPP (SSP>C, P<0.05). In thigh muscles, no significant differences were observed in the percentages of major fatty acids, including linoleic acid, α-linolenic acid and total n-3 and n-6 PUFAs (Table 8). The n-6/n-3 PUFA ratio was also similar and lower than 3:1 in all groups.

**Table 7:** Fatty acid profile of turkey breast muscles, % of total fatty acids.

FA	Experimental group^1^					SEM	*P*
	C	AP	BP	SP	SSP		
C12:0	0.04	0.04	0.04	0.04	0.04	0.001	0.976
C14:0	0.49	0.47	0.47	0.46	0.46	0.005	0.465
C14:1	0.13	0.13	0.12	0.10	0.12	0.005	0.373
C15:0	0.10	0.11	0.11	0.10	0.10	0.001	0.476
C16:0	21.9	21.8	21.4	21.4	21.4	0.105	0.360
C16:1	4.01	4.09	3.73	3.28	3.77	0.154	0.511
C17:0	0.16	0.17	0.18	0.18	0.17	0.004	0.377
C17:1	0.16	0.15	0.15	0.17	0.14	0.005	0.367
C18:0	8.04	7.78	8.06	8.46	7.95	0.152	0.728
C18:1 Cis9	23.5	23.67	22.4	22.2	22.8	0.354	0.648
C18:2 n-6	26.4	27.2	27.1	27.2	26.9	0.231	0.839
C18:3 n-3	9.84^b^	9.60^b^	10.7^a^	10.7^a^	10.9^a^	0.143	0.005
C20:0	0.10	0.11	0.13	0.12	0.10	0.005	0.272
C20:1	0.17	0.17	0.16	0.17	0.16	0.002	0.288
C20:2 n-6	0.27	0.26	0.32	0.27	0.24	0.014	0.473
C20:4 n-6	2.69	2.47	2.87	2.94	2.66	0.110	0.712
C20:5 n-3	0.48	0.41	0.52	0.53	0.49	0.021	0.392
C22:0	0.09	0.09	0.10	0.10	0.09	0.004	0.753
C22:5 n-3	0.96	0.85	1.05	1.07	1.02	0.048	0.621
C22:6 n-3	0.52	0.43	0.44	0.55	0.45	0.028	0.626
SFA	30.9	30.5	30.4	30.8	30.3	0.180	0.845
MUFA	27.9	28.2	26.6	26.0	27.0	0.499	0.602
PUFA	41.2	41.3	43.0	43.25	42.8	0.428	0.390
n-6 PUFA	29.9	30.3	30.8	30.9	30.3	0.309	0.847
n-3 PUFA	11.3^b^	10.9^b^	12.2^a^	12.31^a^	12.4^a^	0.154	0.002
n-6/n-3 PUFA ratio	2.6^b^	2.8^a^	2.5^b^^c^	2.5^b^^c^	2.4^c^	0.026	0.001

^1^Dietary treatments with the addition of: C–cellulose, AP–apple pomace, BP—blackcurrant pomace, SP—strawberry pomace, SSP–seedless strawberry pomace. Values are means of 7 observations per treatment.

^a, b, c^–values marked with different letters in rows differ significantly (P≤0.05). SEM–standard error of the mean.

**Table 8:** Fatty acid profile of turkey thigh muscles, % of total fatty acids.

FA	Experimental group^1^					SEM	*P*
	C	AP	BP	SP	SSP		
C12:0	0.05	0.05	0.05	0.05	0.05	0.001	0.619
C14:0	0.59	0.55	0.58	0.56	0.56	0.006	0.072
C14:1	0.13	0.11	0.13	0.10	0.12	0.005	0.444
C15:0	0.11	0.11	0.12	0.11	0.11	0.002	0.238
C16:0	22.1	21.7	22.2	21.7	22.0	0.133	0.716
C16:1	3.95	3.66	3.94	3.17	3.59	0.171	0.659
C17:0	0.18	0.19	0.20	0.20	0.19	0.004	0.629
C17:1	0.15	0.18	0.16	0.15	0.14	0.006	0.425
C18:0	8.23	8.56	8.42	9.00	8.65	0.149	0.637
C18:1 Cis9	21.8	21.8	22.0	20.6	21.3	0.285	0.597
C18:2 n-6	28.7	28.8	27.8	28.9	28.7	0.243	0.629
C18:3 n-3	10.3	9.90	10.3	10.73	10.2	0.125	0.413
C20:0	0.12	0.12	0.12	0.14	0.12	0.003	0.106
C20:1	0.18	0.18	0.17	0.17	0.17	0.002	0.553
C20:2 n-6	0.21	0.23	0.22	0.24	0.25	0.005	0.302
C20:4 n-6	1.95	2.30	2.21	2.47	2.32	0.076	0.312
C20:5 n-3	0.39	0.39	0.40	0.45	0.41	0.016	0.777
C22:0	0.08	0.09	0.09	0.10	0.09	0.003	0.219
C22:5 n-3	0.56	0.68	0.65	0.75	0.69	0.028	0.381
C22:6 n-3	0.29	0.34	0.26	0.37	0.32	0.016	0.248
SFA	31.4	31.4	31.8	31.9	31.8	0.164	0.845
MUFA	26.2	26.0	26.4	24.2	25.4	0.451	0.304
PUFA	42.3	42.6	41.8	43.9	42.9	0.417	0.640
n-6 PUFA	31.2	31.7	30.6	32.1	31.7	0.297	0.619
n-3 PUFA	11.1	10.9	11.2	11.8	11.2	0.147	0.421
n-6/n-3 PUFA ratio	2.8	2.9	2.7	2.7	2.8	0.026	0.116

^1^Dietary treatments with the addition of: C–cellulose, AP–apple pomace, BP—blackcurrant pomace, SP—strawberry pomace, SSP–seedless strawberry pomace. Values are means of 7 observations per treatment.

SEM–standard error of the mean.

The applied dietary treatments with fruit pomaces affected selected parameters of blood plasma antioxidant status in turkeys (Table 9). The highest vitamin C concentration was noted in group BP, and it differed significantly from those determined in treatments C and SP. Plasma SOD activity was similar in all groups, and catalase activity was highest in groups AP and BP (P<0.05 vs. C and SSP). FRAP levels were highest in groups AP and SSP (P<0.05 vs. C and SP). The highest vitamin A levels in raw breast meat were determined in meat samples collected in group AP (P<0.05 vs. C and BP treatments). In raw thigh meat, differences approaching statistical significance (*P* = 0.065) were noted between group C (lowest level) vs. groups AP, BP and SSP (highest level). Based on vitamin E levels in raw breast meat, the groups were arranged in the following order: C^a^ > AP^b^ > BP^c^, SP^c^, SSP^c^ (P < 0.05).

**Table 9:** Blood plasma antioxidant status and vitamin E and A levels in turkey meat.

	Experimental group^1^					SEM	*P*
	C	AP	BP	SP	SSP		
Blood plasma:							
Vitamin C, μg/L	113^b^	145^a^^b^	202^a^	146^b^	142^a^^b^	9.001	0.049
SOD, U/mL	25.3	25.2	25.2	25.2	25.2	0.027	0.263
Catalase, U/mL	5.39^b^	7.14^a^	7.02^a^	6.15^a^^b^	5.75^b^	0.182	0.002
FRAP, μmol/L	20.4^c^	50.2^a^	43.4^a^^b^	31.3^b^^c^	56.0^a^	2.821	0.001
Breast muscle							
Vitamin E, μg/g	2.56^a^	2.14^b^	1.77^c^	1.64^c^	1.67^c^	0.070	0.000
Vitamin A, μg/g	0.19^b^	0.23^a^	0.19^b^	0.20^a^^b^	0.20^a^^b^	0.004	0.008
Thigh muscle							
Vitamin E, μg/g	1.84	1.78	1.812	1.27	1.79	0.096	0.100
Vitamin A, μg/g	0.16	0.19	0.19	0.17	0.19	0.005	0.065

^1^Dietary treatments with the addition of: C–cellulose, AP–apple pomace, BP—blackcurrant pomace, SP—strawberry pomace, SSP–seedless strawberry pomace. Values are means of 7 observations per treatment. FRAP–ferric reducing ability of plasma; SOD–superoxide dismutase activity.

^a, b, c^–values marked with different letters in a row differ significantly (*P*≤0.05). SEM–standard error of the mean.

The dietary treatments influenced TBARS values which are indicative of the advancement of oxidative processes in raw, frozen and cooked meat (Table 10). The groups were arranged in the following order based on TBARS levels in raw breast meat: C, AP > BP > SP, SSP (P = 0.001). The groups were arranged in a somewhat different order based on the TBARS content of raw thigh meat: C > AP > BP, SP > SSP (P = 0.001). In comparison with group C, significant differences in TBARS levels were not noted in frozen breast and thigh muscles in group AP, whereas the lowest TBARS concentrations were found in frozen meat from group SSP (breast muscle, P<0.05 vs. C, AP; thigh muscle, P<0.05 vs. C, AP, SP). TBARS levels in cooked fresh meat and cooked frozen meat differed from TBARS concentrations in raw and frozen meat, but the distribution of values was similar: TBARS values were highest in group C, significantly lower in group AP and lowest in group SSP.

**Table 10:** Thiobarbituric acid reactive substances (TBARS) levels in turkey thigh and breast meat (nmol MDA/g).

	Experimental group^1^					SEM	*P*
	C	AP	BP	SP	SSP		
TBARS in thigh meat							
fresh raw meat	137^a^	113^b^	98.1^c^	81.0^c^	60.5^d^	4.842	0.001
frozen raw meat	178^a^	173^a^	132^b^^c^	147^b^	117^c^	4.550	0.000
cooked fresh meat	154^a^	136^b^	95.1^c^	78.9^c^^d^	73.6^d^	5.681	0.000
cooked frozen meat	135^a^	110^b^	72.5^c^	58.7^d^	43.9^e^	5.733	0.000
TBARS in breast meat							
fresh raw meat	115^a^	118^a^	102^b^	50.0^c^	45.9^c^	5.631	0.001
frozen raw meat	137^a^	126^a^^b^	97.6^b^^c^	96.3^b^^c^	70.2^c^	6.381	0.004
cooked fresh meat	198^a^	167^b^	134^c^	115^d^	105^d^	6.031	0.001
cooked frozen meat	160^a^	128^b^	100^c^	99.7^c^	87.7^d^	4.453	0.000

^1^Dietary treatments with the addition of: C–cellulose, AP–apple pomace, BP—blackcurrant pomace, SP—strawberry pomace, SSP–seedless strawberry pomace. Values are means of 7 observations per treatment.

^a, b, c, d, e^–values marked with different letters in rows differ significantly (*P*≤0.05). SEM–standard error of the mean.

## Discussion

The content and composition of polyphenols in fruit pomaces are determined by the type of fruit and the amount of compounds that are transferred to juice. Apple pomace contained a wide variety of compounds belonging to different classes of polyphenols, including flavonols, flavan-3-ols and chalcones [17, 25]. Proanthocyanidins generally account for more than half of polyphenols in apple pomace [25]. In the present experiment, proanthocyanidins constituted 80% of the polyphenolic fraction in apple pomace. The total polyphenol content (5.50 mg/g) of apple pomace was somewhat lower than that reported elsewhere [34], and those differences could be attributed to variations in fruit composition and fruit processing technologies.

In our experiment, dried blackcurrant pomace contained 31.0 mg/g total polyphenols, including anthocyanins and proanthocyanidins at a 1:5 ratio. In another study [35], the polyphenol content of dried blackcurrant pomace was determined at 6 mg/g, with an estimated 85% share of anthocyanins. The above findings could suggest that the polyphenol content of berries determined by HPLC can be underestimated if the extraction yield of polymerized proanthocyanidins and the range of the identified fractions are insufficient [36]. In our study, the results of HPLC indicate that the extraction yield of proanthocyanidins was sufficient. It should also be stressed that the blackcurrant pomace used in the present study contained significant amounts of anthocyanins which are susceptible to degradation and are rarely present in pomaces due to their considerable loss during juice production [37].

Other authors demonstrated that anthocyanins [19], free and bound ellagic acid [38] and proanthocyanidins [39] are the major polyphenols in strawberries, whereas flavonols occur in small amounts [38]. The proanthocyanidin content of strawberry pomace can reach 2.5% on a dry weight basis because those compounds are bound to cell wall polysaccharides and are less likely to be transferred to fruit juice [40]. In our study, seedless strawberry pomace contained a wide range of polyphenols (flavone glycosides, anthocyanins, phenolic acids, dihydrochalcones, ellagitannins and proanthocyanidins) whose total content reached 43 mg/g and was similar to the values noted by other authors [18, 41]. The polyphenol content of SP (containing seeds) was 3 times lower, and the composition of polyphenols was limited to ellagitannins, proanthocyanidins and small amounts of flavone glycosides. For this reason, dietary inclusion of SP increased polyphenol levels from 1.27 mg/g in the control diet to 1.48 mg/g in the SP diet and 1.58 mg/g in the SSP diet. The lower polyphenol content of SP can probably be attributed to two factors: the presence of seeds, which are less abundant in polyphenols than fruit flesh, and higher temperature of convection drying than vacuum drying of seedless pomace (70°C). In other study [42], polyphenol levels in dried fruit decreased with an increase in drying temperature.

In this experiment, the differences in the antioxidant activity of fruit pomaces and turkey diets corresponded to the differences in their polyphenol content. The lowest level of antioxidant activity was noted in apple pomace (32 μmol/TE/g), and it was similar to that reported by other authors [42]. The antioxidant capacity of berry pomaces was many times higher than that of apple pomace, proportionally to polyphenol concentrations. Other authors reported high correlations between the polyphenol content of fruit pomaces or extracts and their antioxidant activity [43]. The above observation largely explains the difference in the antioxidant activity of pomaces from native strawberries and seedless strawberries (84.7 vs. 256.4 μmol/TE/g), which could also be attributed to the lower temperature of vacuum drying of SSP than the temperature of convection drying of SP. In a study by Wojdyło *et al* [42], the antioxidant activity of fruit pomace was negatively correlated with drying temperature. The high antioxidant activity of strawberry pomaces and the considerable difference between both types of pomace could also be explained by the presence of ellagitannins which are reportedly highly effective radical scavengers and which effectively inhibit the oxidation of human LDL [44].

The results of previous experiments indicate that crude fiber content is more than 4 times higher in berry pomaces than in apple pomace [35] and that α-linolenic acid has a higher share of the total fatty acid pool [45]. Similar observations were made in this experiment, where a higher content of α-linolenic acid lowered the n-6/n-3 PUFA ratio of blackcurrant and strawberry pomaces (below 3:1) relative to apple pomace (around 10:1). Our results suggest that berry pomaces can be an additional source of PUFAs, in particular linolenic acid, but the amount of oil introduced to turkey diets with 5% fruit pomace was relatively low (around 0.5–0.6%). For this reason, significant differences were not observed in the fatty acid profile of experimental diets.

A higher content of polyphenols in diets enriched with fruit pomace did not affect the final body weights of turkeys or the yields of breast and thigh muscles in the carcass. The above resulted from balanced feed intake and feed conversion as well as similar carcass quality parameters in all groups, which was discussed in a separate study [35]. Other authors demonstrated that a moderate increase in the polyphenol content of diets has no adverse effect on feed intake or the growth rate of birds [14, 46], and performance parameters deteriorated only when the dietary inclusion levels of polyphenols exceeded 2.5 g/kg [46].

In all experimental groups, the n-6/n-3 PUFA ratio in breast and thigh muscles was below 3:1, and remained within the range recommended for the prevention of circulatory system diseases [47]. The above confirms that the addition of linseed oil to bird diets contributes to the health benefits of poultry meat. A similar fatty acid profile was noted in thigh meat, whereas the addition of apple pomace to turkey diets increased the n-6/n-3 PUFA ratio of breast meat relative to berry pomaces and the control group. All diets, including the control treatment, were characterized by a similar n-6/n-3 ratio, but its value was considerably higher in apple pomace than in other pomaces. This could suggest that fatty acids from pomaces are more readily transferred into breast muscles than other dietary fatty acids. Other authors [48] found that linoleic acid is more likely to be accumulated in dark meat, whereas n-3 long-chain fatty acids tend to be deposited in the white meat of chickens. The fat content of white meat from turkey breast is only 1%, and it is significantly lower than in dark meat from thighs, which accumulates most dietary PUFAs [49].

The antioxidant effects of polyphenol-containing byproducts have been rarely studied in poultry, and the reported results are ambiguous. In some experiments, diets with higher levels of polyphenols stimulated the activity of superoxide dismutase and glutathione peroxidase in the blood [13] or increased vitamin E concentrations in the blood of quails [9]. In the present study, the dietary inclusion of different fruit pomaces was accompanied by health-promoting changes in blood plasma vitamin C (in BP treatment), catalase (groups AP and BP) and FRAP (groups AP and SSP) values, pointing to the involvement of different mechanisms. Those effects could be attributed, at least partially, to polyphenolic metabolites that can reach internal tissues, as demonstrated in previous studies conducted on laboratory rodents [50, 51]. The results of experiments with rodents, summarized by Manach *et al* [52], revealed that polyphenols and their metabolites may be detected in a wide range of tissues, including the brain, heart, bones and skin. The tissue distribution of selected polyplenols was investigated in experiments performed on pigs [53, 54, 55] and broilers [56, 57]. Recent studies conducted by members of our research team [50, 51] demonstrated that measurable amounts of strawberry polyphenol metabolites could be found in the blood and urine of rats fed diets containing strawberry pomace, extracts and pure polyphenols (e.g. ellagic acid). In those studies, different groups of metabolites were detected, including urolithin A, nasutin A, urolithin A-glucuronide, nasutin A-glucuronide, iso-nasutin A-glucuronide and ellagic acid dimethyl ether glucuronide (DMEAG). Other authors [58] have recently provided new data about the potent antioxidant activity of urolithin A (UA) derived via microbiota-mediated conversion of strawberry ellagic acid.

In the present study, the vitamin E content of thigh meat was similar in all groups, and vitamin E levels in breast meat were even lower than in the control group. The above could be attributed to the fact that fruit pomaces were more likely to increase the antioxidant activity of the hydrophilic fraction than the lipophilic fraction of turkey diets. According to some authors, there is evidence to suggest that dietary supplements rich in polyphenols can act similarly to dietary vitamin E in the prevention of breast and thigh meat oxidation in chicken, but without any deterioration in tissue reserves of that vitamin [14]. Dietary grape pomace did not influence vitamin E reserves in thigh meat of growing chickens [59]. Other authors [60] demonstrated that the antioxidant activity of polyphenols is determined by α-tocopherol levels in the diet, and that polyphenols can partially replace vitamin E or exert antioxidant effects independently. Recent study showed that dietary polyphenol-rich pomegranate by-product considerably reduced lipid oxidation of broilers meat [61]. Our findings suggest that polyphenols and their metabolites exert antioxidant effects on muscles (refer to lower TBARS values in the meat of turkeys fed SP), therefore vitamin E does not have to be accumulated at high concentrations in the body. In a previous study conducted by members of our research team [62], blackcurrant extract inhibited lipid peroxidation induced by high dietary fat as evidenced by lower concentrations of TBARS in the kidneys and serum of rabbits treated with the extract. The suppression of lipid peroxidation observed in the kidneys of rabbits fed diets enriched with the blackcurrant extract could result from the effects of polyphenolic compounds and their metabolites, which could increase filtration in the kidneys and inactivate free radicals. As demonstrated by Jurgonski *et al*. [62], the compounds present in blackcurrant extract were expected to act beneficially at water–lipid interfaces.

In our study, diets containing fruit pomaces and, consequently, higher levels of polyphenols generally did not increase vitamin A concentrations in turkey meat. Higher levels of vitamin A in breast meat were found only in the group where turkey diets were supplemented with apple pomace. Studies conducted by other authors have shown that the concentrations and metabolism of vitamin A seem to be doubly linked to the antioxidant status of tissues: retinol is involved in antioxidant cell protection [63] and retinol metabolism depends on cellular redox status [64].

In our experiment, a significant (P = 0.001) decrease in TBARS levels was noted in raw, frozen and cooked meat of turkeys whose diets were enriched with fruit pomaces. The decrease was most pronounced in the meat of birds fed seedless strawberry pomace, which is characterized by the highest polyphenol concentrations and the highest antioxidant activity *in vitro*. This result is consistent with previous research, which revealed that dietary antioxidants could be absorbed in the gastrointestinal tract, thus affecting the antioxidant status of poultry [65]. Similar findings, confirmed by a decrease in TBARS concentrations in breast meat, were reported in chickens whose diets were supplemented with grapeseed extract rich in polyphenols [66]. Lower TBARS values in meat were also observed when chickens diets were enriched with tea catechins [67] and a mixture of herbal extracts [56]. Jang *et al*. [56] noted that dietary supplementation with herbal extracts increased the content of polyphenols and their metabolites in chicken meat, which indicates that the analyzed compounds could also influence peripheral tissues and organs. Many studies have shown that dietary polyphenols and products of their metabolism may exert direct antioxidant effects, including radical scavenging activity [68], and affect the gene expression of antioxidant enzymes [69]. Their indirect effects include the activation of the Keap1/Nrf2/ARE pathway, resulting in transcriptional induction of a battery of cytoprotective proteins involved in the synthesis and/or regeneration of direct antioxidants [70, 71]. Such an influence is due to the fact that polyphenols can interact with both lipid and protein components of biological membranes [72] and many of them may “penetrate” both hydrophobic areas (such as the lipid bilayers of cells) and hydrophilic areas (e.g. blood serum). Manach *et al*. [52] stressed the fact that the hydrophobicity of polyphenols is between that of vitamin C (highly hydrophilic) and that of vitamin E (highly hydrophobic). The hydrophobic properties of individual polyphenolic compounds vary, for instance kaempferol is almost twice more hydrophobic than quercetin [72]. This fact could be responsible for the differences in the antioxidant properties of polyphenols present in fruit pomaces.

In our experiment, a decrease in TBARS concentrations in turkey meat was accompanied by a decrease in vitamin E content. This paradox could partially explain the fact that pomace polyphenols contributed mainly to the antioxidant potential of the hydrophilic fraction of turkey diets, and exerted a less profound effect on the lipophilic fraction which is a source of vitamins E and A. Another important consideration is that dietary polyphenols, in particular low-molecular-weight compounds absorbed in the upper digestive tract, are typical xenobiotics that are rapidly removed in metabolic processes [73]. Vitamin E acts as a lignan in the metabolism of xenobiotics, and all forms of vitamin E are able to activate gene expression via the pregnane X receptor (PXR), a nuclear receptor regulating a variety of drug metabolizing enzyme [74]. It has also been found that vitamin E is involved in xenobiotic clearance pathways, and that xenobiotics affect vitamin E concentrations [75]. Polyphenolic compounds can also reduce the synthesis of certain proteins involved in the regulation of vitamin E homeostasis [76]. Therefore, it cannot be excluded that the decrease in vitamin E levels in turkey breast meat, observed in our experiment, could be due to the involvement of this vitamin in the biotransformation (glucuronidation, methylation and sulfation) of polyphenols. Such a phenomenon was not observed in dark thigh meat in any of the dietary treatments. The content of vitamin E was maintained, probably because thigh muscles, compared with breast muscles, are more metabolically active and have more than two-fold higher content of fat rich in PUFAs.

Turkeys selected for a fast growth rate have a higher risk for pale, soft and exudative (PSE) meat. Some authors have suggested the use of color score measurements, particularly lightness (L*) values, in the assessment of risk factors for meat becoming PSE, because L* is well correlated with meat pH and water-holding capacity [77]. It has been reported that an undesired, rapid drop in meat pH results from enhanced post-mortem glycolysis, which may lead to increased protein denaturation and subsequently to a lighter meat color. Based on the values obtained in our study, the analyzed breast muscles from turkeys fed diets containing different fruit pomaces could be considered normal-quality turkey meat.

In conclusion, dried apple, blackcurrant, strawberry and seedless strawberry pomaces differ in the content and composition of polyphenols and antioxidant activity *in vitro*. The highest polyphenol levels and antioxidant activity *in vitro* were determined in seedless strawberry pomace, and the above parameters were lowest in apple pomace. Relatively low inclusion levels (5% of the diet) of fruit pomaces in turkey diets containing linseed oil increased the antioxidant status of blood plasma and decreased TBARS concentrations in raw, frozen and cooked meat in a polyphenol dose-dependent manner. In view of the improved antioxidant stability of turkey meat, fruit pomaces should be considered a valuable delivery vehicle for ingredients which may protect the body against the damaging effects of free radicals.
